# A low dietary sodium dose is associated with a more pronounced aldosterone response in normotensive than in hypertensive individuals

**DOI:** 10.1038/s41598-023-46285-8

**Published:** 2023-11-03

**Authors:** Niels Graudal, Thorbjørn Hubeck-Graudal, Gesche Jurgens

**Affiliations:** 1 Center for Rheumatology and Spine Diseases, The Lupus and Vasculitis Clinic 4242, Copenhagen University Hospital Rigshospitalet, Juliane Maries Vej 10, Copenhagen, Denmark; 2 Department of Nuclear Medicine, Zealand University Hospital, Næstved, Ringstedgade 61, 4700 Næstved, Denmark; 3 Clinical Pharmacology Unit, Zealand University Hospital, Roskilde, Sygehusvej 10, 4000 Roskilde, Denmark

**Keywords:** Cardiology, Endocrinology

## Abstract

In this comprehensive meta-regression analysis encompassing 79 randomized controlled trials, we observed that in populations assigned to a high sodium intake level exceeding 94 mmol, there was no discernible link between plasma aldosterone levels and sodium intake. However, among populations with normal blood pressure subjected to a lower sodium intake, falling below 111 mmol (N = 1544), the association between sodium intake and plasma aldosterone levels manifested as a decrease of 192 pg/ml per 100 mmol of sodium (95% CI − 303 to − 81). In hypertensive populations (N = 1145), this association was less pronounced, with a reduction of 46 pg/ml per 100 mmol sodium, (95% CI − 112 to 20). Furthermore, in normotensive populations the plasma aldosterone increase associated with a decrease in sodium intake was 70 pg/ml per 100 mmol sodium (95% CI 27 to 113). In hypertensive populations, the observed increase was more modest, at 30 pg/ml per 100 mmol sodium, (95% CI 6.8 to 54). A limitation of this study lies in the absence of individual participant data. Our analysis included adjustments for potential effect-modifiers, encompassing bias estimation, which did not substantially alter these associations. One perspective of the present results may be to prompt a reconsideration of current sodium reduction recommendations.

## Introduction

### Description of the condition

The typical sodium intake across global populations ranges from 100 to 200 mmol^1^, exhibiting variations spanning from 7 to 1500 mmol^2^. Sodium homeostasis is primarily governed by the renin–angiotensin–aldosterone system (RAAS). The RAAS is triggered within juxtaglomerular cells in response to reduced blood pressure^3^, activation of the sympathetic nervous system^4^, or stimulation by macula densa cells due to diminished sodium levels in the distal convoluted tubule^5^. In addition to being a key component of the RAAS, aldosterone synthesis is influenced by elevated serum potassium levels^6^, plasma acidosis^7^, and stretch receptors in the heart^8^.

### Historical context of aldosterone

In 1855, Addison characterized clinical manifestations associated with diseases of the suprarenal glands^9^, while later, Brown-Sequard demonstrated that adrenalectomy led to fatality^10^. In 1932, Loeb linked hyponatremia to Addison's disease^11^. In 1935–1936, Kendall et al.^12^ and Reichstein et al.^13^ independently isolated various compounds from bovine adrenal glands, including compound E (cortisone) and compound F (cortisol). In 1937, Reichstein et al. synthesized deoxy-corticosteroid (DOC), a precursor to aldosterone and cortisol^14^. Subsequently, the acetate form of this compound (DOCA) was introduced as a treatment for Addison's disease in 1939^15^. Throughout the 1940s, numerous experiments demonstrated the multifaceted effects of these synthesized hormones and extracts from the adrenal cortex. In 1946, Selye and Jensen classified hormones of the adrenal cortex into glucocorticoids (causing hyperglycemia and glycogen deposition), lipo-corticoids (causing fat deposition, especially in the liver), and mineralocorticoids (causing sodium retention and normalization of subnormal blood sodium and chloride levels in adrenalectomized animals)^16^. Later, the lipo-corticoid effect was linked to glucocorticoid hormones^17^. During this era, while the remarkable impact of cortisone on rheumatoid arthritis was described by Hench et al. in 1949^18^, the notion of a specific mineralocorticoid hormone was a subject of debate. However, in 1952, an unidentified hormone with a potent, almost selective mineralocorticoid effect was isolated^19^ and named electrocortin. In 1953, the structure of this hormone was finally synthesized, and it was subsequently renamed aldosterone^20,21^.

In 1956, Liddle et al. demonstrated that, in contrast to hydrocortisone, aldosterone was not regulated by ACTH but significantly increased during sodium deprivation^22^. Even before the isolation of aldosterone, Deane et al. had shown that renin injection enlarged the zona glomerulosa in the adrenal cortex^23^. The exploration of the renin-angiotensin system reached a milestone in 1957 with the identification of the octapeptide known as angiotensin II^24^. The stimulatory effect of angiotensin II on aldosterone production was established in 1961^25^, finally defining RAAS, a culmination of a development that began in 1898 with the discovery of renin by Tigerstedt and Bergman^26^. In 1971, Coghlan et al. observed that the impact of sodium deprivation on renin and aldosterone appeared weaker in hypertensive individuals compared to normotensives^27^. In 1972, Williams et al. found that an increase in aldosterone secondary to reduced sodium intake consistently correlated with an increase in renin and/or angiotensin II^28^. In the same year, a cross-sectional study involving 219 participants demonstrated an inverse association between plasma renin and aldosterone levels and sodium intake (measured by 24-h urine sodium excretion, a proxy for sodium intake)^29^. Three years later, Oliver et al. revealed a sustained increase in urinary aldosterone excretion in a population with lifelong low sodium intake^30^. Since 1980, numerous randomized controlled trials (RCTs) have substantiated the rise in renin and aldosterone levels associated with reduced sodium intake^31^.

### Description of the intervention and mechanism

The intervention involves altering dietary sodium intake. To ensure high-quality data, we exclusively included RCTs that assigned participants to diets with varying sodium content. Low sodium intake stimulates aldosterone synthesis through the renin-angiotensin pathway, underscoring the importance of sodium deprivation and renin in controlling aldosterone levels. Consequently, modifying sodium intake is expected to influence renin and trigger the subsequent RAAS cascade.

### Importance of the review

Given the reciprocal relationship between dietary salt intake and vascular responses to angiotensin II^32^, the hyperactivity of RAAS due to low salt intake typically does not elevate blood pressure. However, excessive RAAS activity is believed to be a pathophysiological mediator through various mechanisms^33^, and in certain individuals with essential hypertension, low sodium intake fails to alter vascular responses to angiotensin II^34^. Conversely, a high salt intake exceeding 200 mmol, which suppresses RAAS, is associated with increased mortality^35,36^. Therefore, theoretically, the optimal sodium intake may be the lowest level that does not activate RAAS. Hence, it is essential to determine the threshold at which RAAS is activated by sodium intake. A recent meta-regression analysis, based on RCTs, explored the association between a wide range of sodium intakes and corresponding plasma renin activities, revealing a strong inverse correlation between sodium intake and renin levels, especially below 100 mmol, with a potentially more pronounced effect in normotensive individuals than hypertensives^37^. Consequently, the hypothesis is that the relationship between sodium intake and aldosterone would resemble the observed associations between sodium and renin.

### Objective

The aim of this meta-regression analysis is (1) To establish an association between plasma aldosterone levels and sodium intake within a range encompassing very low to very high intakes, as measured by 24-h urinary sodium excretion in participants from RCTs. (2) To identify a potential upper limit of sodium intake below which aldosterone synthesis is stimulated. (3) To investigate the dose–response relationship between sodium intake and plasma aldosterone in study populations comprising normotensive and hypertensive individuals.

## Methods

We registered a protocol in PROSPERO in 2020 (Registration number: CRD42020150355,

https://www.crd.york.ac.uk/prospero/display_record.php?RecordID=150355) and conducted the review compliant with the PRISMA guidelines^38^. Studies were identified from a pool of studies included in a previous meta-analysis (search date March 10th, 2016)^2^. The study identification was updated on March 18th, 2020^37^. On July 6th, 2022, we performed a post-hoc search to identify recent studies that could potentially be included in the analysis.

A comprehensive description of the search strategy, selection criteria, and statistical methods is available in a recent open-access article^37^. In summary, we included RCTs that assigned healthy or untreated hypertensive participants to diets with varying sodium content, provided that sodium intake was measured through 24-h urinary sodium excretion, and plasma aldosterone was assessed as an outcome. Concomitant interventions were allowed unless they were known to impact plasma aldosterone. Our outcome measures included plasma aldosterone levels during low- and high-sodium diets, as well as the mean difference (MD) between these values (indicating the change in aldosterone associated with a reduction in sodium intake). Using plasma aldosterone as the dependent variable, we analyzed the relationship between sodium excretion and plasma aldosterone in study populations randomized to normal/high sodium intake, as well as in those randomized to low sodium intake. We also investigated the relationship between sodium reduction and its effect on plasma aldosterone. We conducted univariable meta-regression analyses and explored the impact of potential effect-modifying covariates (such as study duration, age, SBP, DBP, and weight) through multivariable regression analyses. All analyses were weighted by the inverse variance of the effect on plasma aldosterone, utilizing “R” version 3.6.3^39^.

To assess heterogeneity, we utilized the I^2^ value, expressed as a percentage, as per the method integrated into the forest plot of the Cochrane statistical software (Review Manager Version 5.4, The Cochrane Collaboration, 2020)^40^. An I^2^ value above 75% indicates considerable heterogeneity. We employed a random-effects model to estimate the summary measure as the mean within a distribution of effects^40^. Subgroup analyses were conducted for groups with contrasting bias assessments based on the Cochrane risk of bias tool^40^.

## Results

### Search

A primary search yielded 231 Randomized Controlled Trials (RCTs)^2,37^, with 82 of them measuring plasma aldosterone levels. Subsequently, nine studies were excluded (see Appendix, p. 1) due to reasons outlined therein. The outcomes of these excluded studies were consistent with those of the 73 studies included in the analysis (refer to Appendix reference list 11–83). Six studies provided distinct data for individuals with normal blood pressure and those with hypertension, resulting in the analysis of 79 study interventions. A post-hoc search (Appendix, p. 1) identified one potentially eligible study^41^.

### Study characteristics

Table 1 displays the mean study characteristics and outcomes of meta-analyses concerning the impact of sodium reduction on plasma aldosterone levels in normotensive and hypertensive individuals. For more detailed individual study characteristics, please refer to Appendix Table 1. Notably, the forest plot in Appendix Fig. 1 illustrates individual study outcomes and highlights considerable heterogeneity among the studies, as indicated by high I^2^ values (I^2^ = 96% and I^2^ = 90%). The assessment of bias is presented in Appendix Table 2.

**Table 1:** Study characteristics and outcomes of meta-analyses of the effect of sodium reduction on plasma aldosterone in normotensive and hypertensive individuals.

	Normal blood pressure	Hypertension
N, trials	48	31
N, participants	1544	1145
Mean Age (range), years	32 (13–64)	49 (27–73)
Mean study duration (range), days	8 (3–42)	14 (4–42)
Mean baseline SBP (range) mmHg	118 (107–140)	152 (120–176)
Mean baseline DBP (range) mmHg	70 (56–84)	94 (75–108)
Mean 24-h high sodium (range), mmol	218 (111–386)	207 (94–370)
Mean 24-h low sodium (range), mmol	42 (8–105)	55 (13–111)
Mean sodium reduction (range), mmol	176 (42–341)	152 (55–331)
Mean effect, aldosterone (95% CI), pg/ml*	142.5 (118.3–166.7)**	74.5 (60.4–88.7)**
N, trials with significant effect^#^	41	24
N, trials with opposite effect^##^	0	0

*Mean increase in pl-aldosterone (picogram/ml) associated with sodium reduction.

**p < 0.00001.

^#^Number of individual trials with a significant increase in pl-Aldosterone associated with sodium reduction.

^##^Number of individual trials with a reduction in pl-Aldosterone associated with sodium reduction.

### Sodium–aldosterone relationship

The analysis revealed a statistically significant increase in the mean plasma aldosterone during low sodium intake (as depicted in Table 1). Specifically, the increase was notably higher in study populations with normal blood pressure (142.5 pg/ml) compared to those with hypertension (74.5 pg/ml) (t = 4.3 and p < 0.00001).

**Figure 1:**
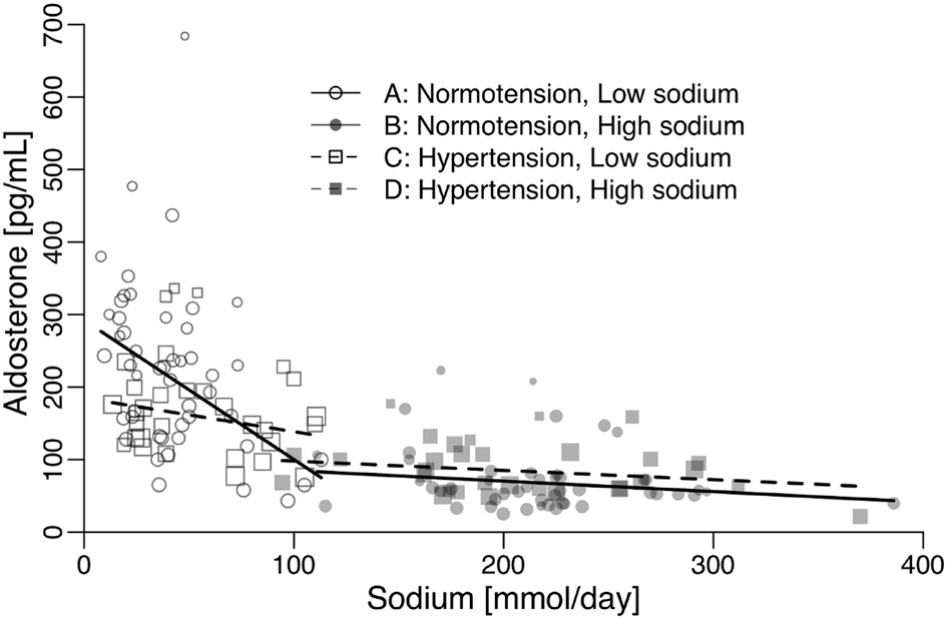
The relationship between the mean plasma-aldosterone and mean sodium-intake/day assessed as 24-h urinary sodium-excretion in 48 studies of normotensive individuals and 31 studies of untreated hypertensive individuals.

In populations with normal blood pressure, we observed a significant association between plasma aldosterone levels and sodium intake during periods of low sodium intake (− 192 pg/ml per 100 mmol of sodium; 95% CI − 303 to − 81; p = 0.001), as illustrated in Fig. 1A and detailed in Table 2A. A similar trend was observed in populations with hypertension, although it did not reach statistical significance (− 46 pg/ml per 100 mmol of sodium; 95% CI − 112 to 20; p = 0.17), as shown in Fig. 1C and explained in Table 2B. Notably, we found no significant associations between plasma aldosterone levels and the duration of sodium intake, age, systolic blood pressure (SBP), diastolic blood pressure (DBP), or weight in either group, as outlined in Table 2A,B.

**Table 2:** Univariate weighted meta-regression analysis of plasma-aldosterone versus 24-h urinary sodium-excretion and effect-modifiers (duration, age, SBP, DBP and weight).

	Coefficient	2.5%	97.5%	p
A: Low sodium intake. Normotension. Association with plasma-aldosterone (weighted). N = 48				
Sodium intake (low)	− 1.92	− 3.03	− 0.81	0.001
Duration	− 3.81	− 8.07	0.45	0.08
Age	− 1.35	− 4.31	1.61	0.36
SBP (low sodium)	− 3.49	− 8.78	1.79	0.19
DBP (low sodium)	− 2.51	− 7.55	2.52	0.32
Weight (kg)	− 0.41	− 5.21	4.38	0.86
B: Low sodium intake. Hypertension. Association with plasma-aldosterone (weighted). N = 31				
Sodium intake (low)	− 0.46	− 1.12	0.20	0.17
Duration	− 1.00	− 3.02	1.02	0.32
Age	− 0.61	− 3.26	2.04	0.64
SBP (low sodium)	− 1.49	− 3.38	0.41	0.12
DBP (low sodium)	− 0.43	− 3.11	2.25	0.74
Weight (kg)	− 1.00	− 4.35	2.35	0.55
C: Usual/high sodium intake. Normotension. Association with plasma-aldosterone (weighted). N = 48				
Sodium intake (high)	− 0.14	− 0.39	0.10	0.23
Duration	0.77	− 0.88	2.43	0.35
Age	− 0.41	− 1.53	0.72	0.47
SBP (high sodium)	− 0.11	− 1.78	1.56	0.90
DBP (high sodium)	− 1.11	− 2.90	0.69	0.22
Weight (kg)	0.99	− 0.87	2.85	0.29
D: Usual/high sodium intake. Hypertension. Association with plasma-aldosterone (weighted). N = 31				
Sodium intake (high)	− 0.13	− 0.32	0.06	0.18
Duration	0.87	− 0.23	1.96	0.12
Age	− 0.63	− 2.08	0.83	0.39
SBP (high sodium)	0.33	− 0.81	1.47	0.56
DBP (high sodium)	1.52	0.10	2.94	0.04
Weight (kg)	1.24	− 0.69	3.16	0.20
E: Change in sodium intake. Normotension. association with plasma-aldosterone difference (weighted). N = 48				
Sodium reduction	0.70	0.28	1.13	0.002
Duration	− 4.59	− 8.22	− 0.96	0.01
Age	− 0.94	− 3.55	1.67	0.47
SBP (high sodium)	− 3.90	− 8.04	0.23	0.06
DBP (high sodium)	− 2.44	− 7.16	2.29	0.30
Weight (kg)	− 1.40	− 5.70	2.89	0.51
F: Change in sodium intake. Hypertension. association with plasma-aldosterone difference (weighted). N = 31				
Sodium reduction	0.30	0.07	0.54	0.013
Duration	− 1.98	− 3.66	− 0.30	0.022
Age	− 0.23	− 2.61	2.14	0.84
SBP (high sodium)	− 0.92	− 2.73	0.89	0.31
DBP (high sodium)	− 1.05	− 3.59	1.49	0.41
Weight (kg)	− 2.41	− 5.34	0.52	0.10

In the context of normal/high sodium intake, no significant associations were observed between plasma aldosterone levels and factors such as sodium intake, the duration of sodium intake, age, systolic blood pressure (SBP), diastolic blood pressure (DBP), or weight. This lack of association was consistent across both study populations with normal blood pressure and those with hypertension (Fig. 1B,D, and Table 2C,D).

**Figure 2:**
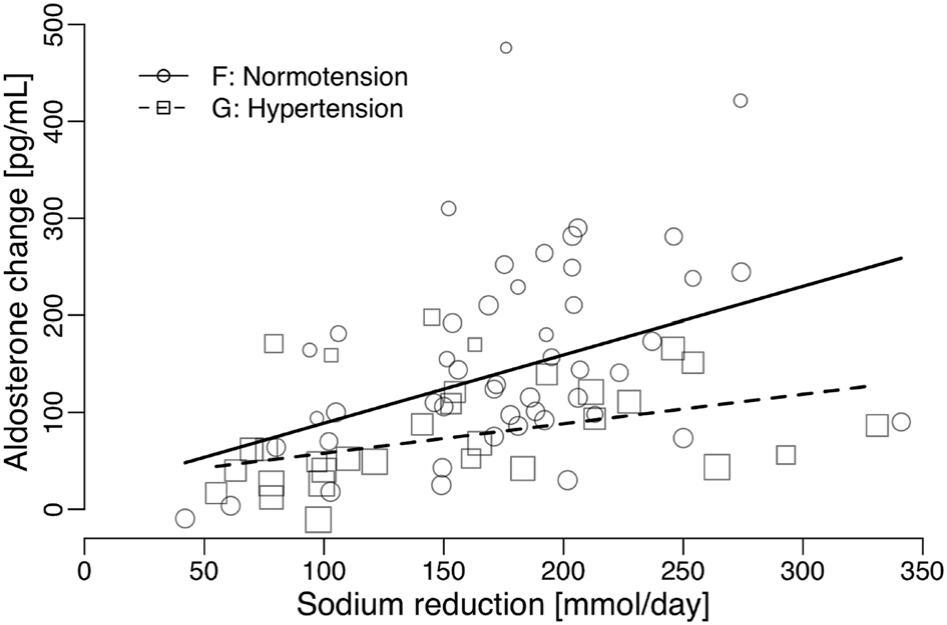
The relationship between the change in mean plasma-aldosterone and the change in mean sodium-intake/day assessed as 24-h urinary sodium-excretion in 48 studies of normotensive individuals and 31 studies of untreated hypertensive individuals.

However, when the transition was made from normal/high sodium intake to low sodium intake, there was a notable correlation between the change in plasma aldosterone levels and the corresponding change in sodium intake. In individuals with normal blood pressure, this relationship was statistically significant, showing an increase of 70 pg/ml per 100 mmol decrease in sodium intake (95% CI 27–113, p = 0.0018), as detailed in Table 2E and illustrated in Fig. 2F. Similarly, among individuals with hypertension, a significant association was observed, with a rise of 30 pg/ml per 100 mmol decrease in sodium intake (95% CI 6.8–54, p = 0.01), as outlined in Table 2F and depicted in Fig. 2G. Furthermore, the change in plasma aldosterone was also linked to the duration of sodium intake, as shown in Table 2E,F, However, there were no discernible associations between plasma aldosterone change and age, SBP, DBP, or weight, as summarized in Table 2E,F.

**Figure 3:**
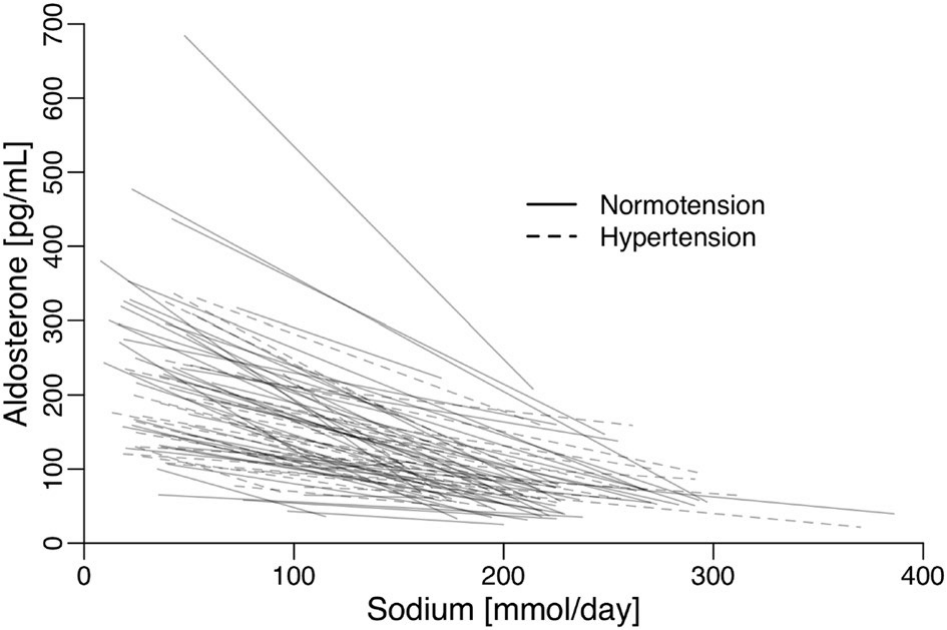
The within-study relationship between the change in plasma-aldosterone and the change in sodium-intake/day assessed as 24-h urinary sodium-excretion in 48 studies of normotensive individuals and 31 studies of untreated hypertensive individuals.

Figure 3 illustrates the correlation between changes in plasma aldosterone and sodium intake for each individual study, while Table 3 presents the results of the multivariable analyses examining the relationship between plasma aldosterone and potential effect modifiers.

**Table 3:** Multivariate weighted meta-regression analysis of plasma-aldosterone versus 24-h urinary sodium-excretion, age and SBP.

	Estimate	2.5%	97.5%	P
A: Low sodium intake. Normotension. Association with plasma-aldosterone (weighted). N = 48				
Sodium intake (low)	− 1.84	− 3.33	− 0.34	0.02
Age	0.30	− 2.82	3.41	0.85
SBP (low sodium)	0.17	− 5.78	6.13	0.95
B: Low sodium intake. Hypertension. Association with plasma-aldosterone (weighted). N = 31				
Sodium intake (low)	− 0.34	− 1.05	0.36	0.32
Age	0.21	− 2.60	3.01	0.88
SBP (low sodium)	− 1.26	− 3.42	0.90	0.24
C: Usual/high sodium intake. Normotension. Association with plasma-aldosterone (weighted). N = 48				
Sodium intake (high)	− 0.10	− 0.34	0.14	0.41
Age	− 0.49	− 1.66	0.68	0.40
SBP (high sodium)	0.10	− 1.76	1.96	0.91
D: Usual/high sodium intake. Hypertension. Association with plasma-aldosterone (weighted). N = 31				
Sodium intake (high)	− 0.15	− 0.34	0.04	0.11
Age	− 1.39	− 3.05	0.28	0.10
SBP (high sodium)	0.82	− 0.45	2.09	0.19
E: Change in sodium intake. Normotension. Association with plasma-aldosterone (weighted). N = 48				
Sodium reduction	0.65	0.16	1.13	0.01
Age	− 0.32	− 3.02	2.38	0.81
SBP (high sodium)	− 1.76	− 6.32	2.79	0.44
F: Change in sodium intake. Hypertension. Association with plasma-aldosterone (weighted). N = 31				
Sodium reduction	0.31	0.07	0.56	0.01
Age	1.09	− 1.47	3.65	0.39
SBP (high sodium)	− 1.08	− 3.03	0.87	0.27

Recognizing the collinearity issue between SBP (Systolic Blood Pressure) and DBP (Diastolic Blood Pressure), we aimed to include only one of these variables. However, our analysis did not reveal any discernible patterns in the associations, as depicted in Table 2. Consequently, we made an arbitrary decision to incorporate SBP in the primary analysis, as detailed in Table 3.

Weight exhibited a weak association with plasma aldosterone in the univariable analyses (Table 2). Nevertheless, since weight data were not consistently available across all studies, we opted to exclude it from the model. Additionally, due to the significant collinearity observed with sodium intake, we also excluded the duration of sodium intake from the model, as indicated in Ref.^37^.

It's worth mentioning that when DBP was used as an effect modifier instead of SBP, the associations between plasma aldosterone and sodium intake remained similar, as shown in Appendix Table 3.

Lastly, a recent study^41^, which was not included in the present analysis, yielded results consistent with those of all other studies. Specifically, it indicated an increase in plasma aldosterone levels during periods of low sodium intake.

## Discussion

### Summary of key findings

The sodium reductions observed in this study align with global recommendations for reducing sodium intake to below 100 mmol/day by the WHO^42^ and below 65 mmol/day by the American Heart Association^43^.

In populations with high sodium intake (above 94 mmol for hypertensives and 111 mmol for normotensives), plasma aldosterone remains stable, and levels are similar between normotensive and hypertensive individuals (Fig. 1). In populations with lower sodium intake (below 111 mmol for hypertensives and 105 mmol for normotensives), an inverse relationship between sodium intake and plasma aldosterone is observed (Fig. 1). Reducing sodium intake from a high level (above 94–111 mmol) to a low level (below 105–111 mmol) is associated with an increase in plasma aldosterone (Fig. 2). This increase seems more pronounced with lower target sodium intake levels (Fig. 3) and is stronger in normotensive individuals compared to hypertensive individuals (Figs. 1 and 2, Tables 2 and 3). The multivariable coefficients (Table 3) and univariable coefficients (Table 2) were similar. Thus, interactions between effect-modifiers seem negligible.

### Overall completeness and applicability of evidence

Overall, this study comprehensively investigated the association between sodium intake and aldosterone in both healthy normotensive individuals and untreated hypertensive individuals, covering a wide range of sodium intake levels (100–400 mmol) common in the general population. Additionally, it highlights the impact of sodium intake below 100 mmol complying with health recommendations^42,43^.

### Quality of evidence

The study maintained a high level of evidence by including only randomized controlled trials (RCTs). Despite considerable heterogeneity, the evidence remains strong, with all studies consistently demonstrating an increase in plasma aldosterone when transitioning from high to low sodium intake levels (Table 1, Fig. 3, Appendix Fig. 1). The substantial number of studies (n = 79) and participants (1544 normotensive and 1145 hypertensive individuals) adds to the robustness of the analyses.

### Potential biases and limitations

One limitation is the lack of separate data for females and males in the RCTs, preventing the analysis of potential gender differences. The uniform and extreme reduction in sodium intake may also be seen as a limitation, but the variability in sodium reduction within the study population was significant (ranging from about 50 to 350 mmol).

Most studies had a duration of less than 2 weeks, which could be considered a limitation. However, an observational study of Yanomamo Indians living with a constant low-sodium diet throughout their lives demonstrated a significant 15-fold increase in urinary excretion of aldosterone^30^. This study concludes as follows: “Simultaneous plasma-renin activities were elevated and comparable to those of civilized subjects placed for brief periods on 10 mEq sodium diets. Similarly, excretion rates of aldosterone equaled those of acculturated subjects on low sodium diets. The findings suggest that the hormonal adjustments to life-long low sodium intakes are similar to those achieved in acute sodium restriction of civilized man”.

In populations with low sodium intake, aldosterone was inversely associated with study duration (Table 2A,B), likely due to the direct relationship between study duration and sodium intake as shown previously^37^. Similarly, the inverse association between duration and the change in aldosterone associated with the change in sodium intake (Table 2E,F) probably is due to the inverse association between duration and the change in sodium-intake^37^. Duration was directly associated with plasma-aldosterone but was also associated with sodium intake. Due to this collinearity, we assume that this association is not independent. The other potential effect modifiers did not show significant associations with aldosterone, suggesting negligible contribution to heterogeneity.

The study assumes that laboratories measuring 24-h urinary sodium excretion and plasma aldosterone were independent and blinded to sodium intake levels, resulting in low risk of detection bias.

A limitation is that studies of hypertensive individuals did not investigate primary aldosteronism. Thus, an unknown fraction of participants may have a blunted rise in aldosterone during sodium restriction compared with the renin rise, as the aldosterone secretion partially is autonomous in these patients^44^.

The study excluded RCTs that included treated hypertensive patients, thus avoiding bias from antihypertensive treatments affecting plasma aldosterone. For instance, aldosterone is suppressed in patients treated with ACE inhibitors and angiotensin II antagonists^45^, aldosterone inhibitors^46^ and beta-blockers^47^.

Though statistically significant, the influence of sodium intake on plasma sodium is small^48^. Furthermore, it has been indicated that moderate variations in plasma-sodium is not associated with plasma-aldosterone^47^, indicating that the effect of dietary sodium on aldosterone is likely not mediated via changes in plasma sodium concentration.

A high dietary sodium intake, however, leads to reduced sodium reabsorption in the proximal tubule, resulting in an increased sodium load in distal segments. Conversely, a low dietary sodium intake enhances sodium reabsorption in the proximal tubule, reducing the sodium load in distal kidney tubule segments^49^. The synthesis of aldosterone is stimulated by a low sodium concentration in these distal segments, explaining why a low sodium diet triggers increased aldosterone production. In contrast, under pathological conditions, a high sodium intake can significantly elevate aldosterone levels, plasma sodium concentration, and blood pressure (BP).

In cases where high salt-induced activation of collecting duct nitric oxide synthase 1b signaling fails, it can lead to the suppression of the systemic and intrarenal renin–angiotensin–aldosterone system, resulting in an increase in plasma sodium and BP^50^. This mechanism has been proposed as a cause of salt-sensitive hypertension^51^. Other genetic factors contributing to salt-sensitive hypertension mediated by aldosterone have also been identified. For instance, during the transition from a low to high sodium diet, individuals with the 46AA/79CC beta-2 adrenergic receptor diplo-type experience a more significant increase in blood pressure and aldosterone levels, along with lower plasma renin and serum potassium levels. This suggests the involvement of the beta-2 adrenergic receptor in the regulation of aldosterone secretion. These findings were further confirmed in isolated zona glomerulosa cells, where stimulation of the beta-2 adrenergic receptor increased aldosterone secretion, while its blockade reduced the stimulated aldosterone response^52^. In rat studies, it has also been indicated that calorie restriction may increase aldosterone by enhancing the response of zona glomerulosa cells to angiotensin II^53^.

While these factors may have the potential to introduce bias in trial outcomes, they are relatively rare and are expected to be randomly distributed in randomized controlled trials (RCTs). Therefore, it is unlikely that they significantly impact the results of the present analysis.

### Agreements and disagreements with other studies

In addition to the RCTs analyzed in this study, experimental and observational studies have found similar inverse associations between sodium intake and plasma aldosterone in individual patients. However, these studies did not investigate separate effects above and below 100 mmol sodium intake or separate effects in hypertensive and normotensive individuals^29,54^.

In one study, sodium intake was reduced to an average level of 84 mmol, resulting in a slight yet statistically significant 10% increase in aldosterone^55^. Conversely, in studies that reduced sodium intake to levels as low as 10–20 mmol, aldosterone surged by 300–500%^56–60^, aligning closely with the outcomes observed in our study.

Plasma aldosterone responses appear stronger in normotensive populations than in hypertensive ones (Fig. 2). Coghlan et al. reported analogous results, noting that plasma aldosterone levels did not exhibit a distinction between normotensive and hypertensive individuals during sodium loading. However, during sodium restriction, the increase in urinary aldosterone secretion was notably more pronounced in normotensive subjects compared to hypertensive ones^27^. In alignment with our prior observations^37^, Coghlan et al. also documented a diminished rise in renin levels among hypertensive participants.

The intake of 100 mmol of sodium appears to represent a physiologically significant threshold. Below this level, both plasma aldosterone and plasma renin levels tend to increase. This observation may help explain why the typical sodium intake in many global populations exceeds 100 mmol^1^. It also sheds light on why reducing sodium intake has only modest effects on blood pressure^2,31^ and why achieving sodium levels below 130 mmol in long-term clinical trials has proven challenging^61^. Additionally, elevated plasma aldosterone levels have been linked to higher mortality rates^33,62–64^, which partially accounts for the increased mortality risk associated with low sodium intake^35,36^, or at the very least, the absence of clear benefits^65,66^. These findings do not lend strong support to the idea of universally reducing dietary sodium intake to levels below 100 mmol. However, certain specific patient groups may still benefit from such an intervention^67^.

In summary, our study confirms that reducing sodium intake is linked to a proportional increase in plasma aldosterone levels. We have identified a critical threshold of around 100 mmol of sodium intake below which this association becomes more apparent. Notably, this connection seems to be more pronounced in healthy individuals compared to those with hypertension.

### Supplementary Information

Supplementary Information.

## Data Availability

All data generated or analyzed during this study are included in the supplementary information files of the published article. Additional data is available from the corresponding author on reasonable request.
