# Supplementary material for: A low dietary sodium dose is associated with a more pronounced aldosterone response in normotensive than in hypertensive individuals
Source: Sci Rep. 2023 Nov 3;13:19027. doi: 10.1038/s41598-023-46285-8 (PMC10624927; doi:10.1038/s41598-023-46285-8)
Supplement: Supplementary file 1 — Supplementary Information. [file 41598_2023_46285_MOESM1_ESM.docx]

# **Appendix: A low dietary sodium dose is associated with a more pronounced aldosterone response in normotensive than in hypertensive individuals**

Niels Graudal, Thorbjørn Hubeck-Graudal, Gesche Jürgens,

**Results**

*Reasons for exclusion of studies:* From the identified pool of 231 RCT studies (1) we selected 82 studies, which measured aldosterone as outcome. Three studies (2-4) were excluded due to use of anti-hypertensive medications. Five parallel trials were excluded (5-9). One outlier study did not reduce sodium intake from a high level to a low level but from a very high level (357 mmol) to another very high level (310 mmol) and was therefore also excluded from the analysis (10). Thus 73 references (11-83) were integrated in the statistical analyses.

*Post-hoc search:* On July 6^th^ 2022 we performed a last additional search to record potentially eligible studies, which might have been published during the preparation of our manuscript and therefore not included in the present analysis.

We used a broad search term in PubMed (Medline and additional databases), “sodium and aldosterone” giving 14041 results, then using publication date January 1^st^, 2020 (overlapping our official search date, March 18^th^, 2020) as a filter to cover the period January 1^st^, 2020 - July 6^th^, 2022, giving 684 results. We identified five RCTs from this pool, of which two were in patients with chronic kidney disease (84-85) and one was in patients with diabetes (86). In one study sodium reduction was not achieved (87). Thus, none of these 4 studies were eligible. One study, published online January 2021, included healthy young males and showed, as all other studies that aldosterone increased during the low sodium diet (88) (ref 19 in the manuscript).

*Risk of bias across studies:* In populations with usual/high sodium intake there was significant inverse collinearity and in populations with low sodium intake there was a significant direct collinearity between sodium intake and duration of sodium intake (1). In populations with usual/high sodium intake there was an inverse collinearity between sodium reduction and duration of sodium intake in normotensive and hypertensive studies (1).

The difference in pl-aldosterone between usual/high sodium intake and low sodium intake did not differ between normotensive studies with low risk of bias (109 pg/ml (54-164)) and high/unclear risk of bias (146 pg/ml(119-172)), p = 0.57, or between hypertensive studies with low risk of bias (69 pg/ml (53-85)) and high/unclear risk of bias (96 pg/ml(51-140)), p = 0.24. The difference in pl-aldosterone between usual/high sodium intake and low sodium intake did not differ between normotensive studies on diet (137 pg/ml (108-166)) and sodium tablet (149 pg/ml (105-193)), p = 0.66, or between hypertensive studies on diet (110 pg/ml (64-156)) and sodium tablet (69 pg/ml (54-84)), p = 0.07.

*Certainty* *(quality of evidence)*: This was estimated previously to be high (89).

**Appendix Table 1:** Characteristics of included studies

| Ref  no | First Author | Dur.  (days) | Restric  Method | BP | N | Age | Weight  (kg) | High Sod.(HS) | Los Sod. (LS) | SR | SBP (H) | DBP (H) | SBP (L) | DBP (L) |
| --- | --- | --- | --- | --- | --- | --- | --- | --- | --- | --- | --- | --- | --- | --- |
| 11 | Sullivan | 4 | diet | N | 27 | 28,8 | 68,1 | 170,00 | 24,00 | 146 | 107,0 | 59,60 | 113,90 | 60,70 |
| 11 | Sullivan H | 4 | diet | H | 19 | 27 | 76,8 | 178,00 | 25,00 | 153 | 120,3 | 74,70 | 121,50 | 73,50 |
| 12 | Skrabal | 14 | diet | N | 20 | 23 | 75,8 | 200,00 | 50,00 | 150 | 125,0 | 73,10 | 122,30 | 70,10 |
| 13 | Mcgregor | 28 | ss | H | 19 | 49 | 80,1 | 162,00 | 86,00 | 76 | 156,0 | 98,01 | 146,01 | 93,01 |
| 14 | Sowers | 6 | diet | N | 9 | 39 | NA | 196,00 | 42,40 | 153,6 |  |  |  |  |
| 15 | Skrabal | 14 | diet | N | 52 | 23 | 76,5 | 194,00 | 38,00 | 156 | 121,1 | 63,60 | 117,96 | 61,75 |
| 16 | Koolen | 14 | ss | H | 20 | 40,8 | 76,9 | 270,00 | 57,00 | 213 | 147,7 | 95,40 | 141,51 | 90,50 |
| 17 | Richards | 28 | ss | H | 12 | 36 | 74,3 | 180,00 | 80,00 | 100 | 149,9 | 92,40 | 145,92 | 89,40 |
| 18 | Richards | 4 | diet | N | 8 | 36 | NA | 193,00 | 12,00 | 181 | 110,0 | 56,00 | 108,00 | 63,00 |
| 19 | Shore | 5 | ss | H | 6 | 51,9 | 77,3 | 122,00 | 25,00 | 97 | 157,0 | 101,01 | 148,02 | 95,41 |
| 20 | Hargreaves | 14 | ss | N | 8 | 23,4 | 71,4 | 155,00 | 49,00 | 106 | 129,0 | 66,02 | 123,00 | 63,02 |
| 21 | McGregor | 30 | ss | H | 20 | 57 | 73,6 | 190,00 | 49,00 | 141 | 163,0 | 100,02 | 147,00 | 91,02 |
| 22 | Brun | 4 | ss | N | 10 | 46 | 72,2 | 386,00 | 45,00 | 341 | 116,0 | 69,03 | 111,01 | 68,03 |
| 22 | Brun H | 4 | ss | H | 12 | 47 | 77,2 | 370,00 | 39,00 | 331 | 150,0 | 96,01 | 142,01 | 92,01 |
| 23 | Sharma | 7 | ss | N | 15 | 24 | 74,4 | 211,00 | 19,00 | 192 | 106,5 | 68,30 | 105,60 | 64,60 |
| 24 | Sharma | 6 | ss | N | 23 | 25 | 75,4 | 267,00 | 21,00 | 246 | 119,6 | 74,40 | 115,10 | 72,20 |
| 25 | Gow | 7 | diet | N | 9 | 30,3 | NA | 111,00 | 17,00 | 94 | 120,0 | 68,00 | 112,01 | 65,00 |
| 26 | Huggins | 14 | ss | N | 9 | 25 | 71,0 | 170,00 | 73,00 | 97 | 112,0 | 69,01 | 111,01 | 67,01 |
| 27 | Benetos | 28 | ss | H | 20 | 41,5 | 71,6 | 163,00 | 85,00 | 78 | 149,1 | 93,20 | 142,60 | 89,50 |
| 28 | Ruppert | 7 | ss | N | 163 | 38 | 70,7 | 290,90 | 16,70 | 274,2 | 113,2 | 71,50 | 111,00 | 72,50 |
| 29 | Burnier | 6 | diet | N | 23 | 29 | NA | 237,40 | 35,70 | 201,7 | 111,7 | 72,20 | 110,70 | 73,00 |
| 30 | Donovan | 5 | diet | N | 8 | 36 | 67,0 | 160,00 | 8,00 | 152 | 116,0 | 63,00 | 114,00 | 64,00 |
| 31 | Fotherby | 35 | ss | H | 17 | 73 | 67,4 | 174,00 | 95,00 | 79 | 176,0 | 96,04 | 168,00 | 97,04 |
| 32 | Zoccali | 7 | ss | H | 15 | 45 | 79,6 | 217,00 | 54,00 | 163 | 144,0 | 92,01 | 130,02 | 84,01 |
| 33 | Overlack | 7 | ss | H | 46 | 45,3 | 80,5 | 265,40 | 19,70 | 245,7 | 138,0 | 90,20 | 134,10 | 87,70 |
| 34 | Schorr | 28 | ss | N | 16 | 64,1 | 73,9 | 166,00 | 105,00 | 61 | 140,0 | 84,01 | 139,00 | 84,01 |
| 35 | Bellini | 14 | ss | H | 43 | 45,4 | 78,0 | 231,80 | 110,70 | 121,1 | 159,8 | 103,60 | 155,90 | 98,70 |
| 36 | Ferri | 14 | ss | H | 61 | 47,1 | 78,2 | 291,00 | 26,90 | 264,1 | 168,7 | 107,70 | 161,30 | 104,20 |
| 37 | Ishimitsu | 7 | diet | N | 7 | 53 | 56,8 | 217,00 | 22,00 | 195 | 116,0 | 77,00 | 114,04 | 75,00 |
| 37 | Ishimitsu H | 7 | diet | H | 23 | 55 | 59,7 | 217,00 | 24,00 | 193 | 157,2 | 94,50 | 141,50 | 89,00 |
| 38 | Foo | 6 | ss | N | 18 | 51,1 | 71,9 | 227,10 | 77,70 | 149,4 | 122,8 | 76,90 | 115,10 | 79,30 |
| 39 | Damascen. | 7 | ss | N | 20 | 37,5 | 73,0 | 293,00 | 39,00 | 254 | 108,5 | 67,70 | 109,00 | 68,00 |
| 39 | Damascen. H | 7 | ss | H | 19 | 42,5 | 75,0 | 293,00 | 39,00 | 254 | 144,7 | 90,80 | 136,20 | 85,40 |
| 40 | Schorr | 7 | ss | N | 187 | 25 | 73,0 | 225,00 | 19,00 | 206 | 110,7 | 58,00 | 110,50 | 58,30 |
| 41 | Chiolero | 7 | ss | H | 38 | 43 | 75,4 | 255,00 | 72,00 | 183 | 143,1 | 89,80 | 136,60 | 85,60 |
| 42 | Bruun | 4 | diet | N | 42 | 26 | 72,6 | 273,00 | 36,00 | 237 | 117,0 | 70,02 | 117,01 | 71,02 |
| 43 | Burnier | 7 | ss | N | 15 | 22,7 | 72,3 | 207,00 | 18,70 | 188,3 | 126,0 | 75,01 | 125,00 | 80,01 |
| 44 | Cuzzola | 14 | ss | H | 19 | 47 | 77,1 | 261,30 | 99,90 | 161,4 | 153,8 | 97,80 | 148,70 | 97,90 |
| 45 | Manunta | 14 | ss | H | 20 | 48,3 | 79,6 | 176,60 | 66,60 | 110 | 152,4 | 99,20 | 147,20 | 95,90 |
| 46 | Kleij | 7 | diet | N | 27 | 23,7 | 74,1 | 236,00 | 50,00 | 186 | 118,5 | 74,10 | 118,70 | 74,22 |
| 47 | Kerstens | 7 | diet | N | 28 | 23,3 | 73,6 | 248,00 | 42,00 | 206 | 114,5 | 71,51 | 117,60 | 73,51 |
| 48 | Perry | 5 | ss | N | 15 | 26 | 74,0 | 175,00 | 70,00 | 105 | 115,0 | 60,00 | 115,00 | 58,00 |
| 49 | Palacios | 21 | diet | N | 23 | 12,8 | 56,0 | 115,00 | 35,00 | 80 | 112,7 | 56,50 | 115,10 | 57,90 |
| 50 | Beeks | 7 | diet | H | 117 | 53,4 | 80,4 | 171,00 | 72,00 | 99 | 166,0 | 94,00 | 167,20 | 92,20 |
| 51 | Zanchi | 7 | diet | N | 9 | 25 | 70,6 | 270,00 | 20,00 | 250 | 117,0 | 72,00 | 114,02 | 72,00 |
| 52 | Swift | 28 | ss | H | 40 | 50 | 79,0 | 167,00 | 89,00 | 78 | 156,0 | 100,01 | 148,02 | 97,01 |
| 53 | Townsend | 6 | ss | N | 18 | 30 | 69,6 | 194,00 | 23,00 | 171 | 117,0 | 69,04 | 111,00 | 65,04 |
| 54 | Dengel | 8 | diet | H | 28 | 63 | 81,3 | 191,00 | 36,40 | 154,6 | 152,0 | 79,02 | 142,00 | 75,02 |
| 55 | Tzemos | 5 | ss | N | 16 | 27 | 76,8 | 225,00 | 76,00 | 149 | 121,0 | 71,02 | 117,00 | 70,02 |
| 56 | He W | 42 | ss | H | 169 | 50 | 85,3 | 165,00 | 110,00 | 55 | 146,0 | 90,01 | 141,20 | 87,81 |
| 57 | Carey | 7 | diet | N | 185 | 47 | 70,3 | 221,40 | 17,70 | 203,7 | 124,0 | 74,20 | 119,91 | 73,30 |
| 57 | Hyperpath | 7 | diet | H | 211 | 49,2 | 76,2 | 225,00 | 13,50 | 211,5 | 147,5 | 88,80 | 131,50 | 79,50 |
| 58 | Graffe | 4 | diet | N | 21 | 26 | 77,8 | 213,00 | 41,00 | 172 | 124,0 | 76,00 | 126,02 | 77,00 |
| 59 | Krikken | 7 | diet | N | 65 | 23 | 81,0 | 228,00 | 36,00 | 192 | 114,5 | 71,52 | 113,21 | 70,72 |
| 60 | Mallamaci | 14 | ss | H | 32 | 48 | 74,0 | 193,00 | 28,00 | 165 | 143,1 | 91,00 | 135,05 | 88,00 |
| 61 | Dickinson | 42 | diet | N | 25 | 35,1 | 91,0 | 155,00 | 113,00 | 42 | 123,0 | 72,02 | 121,00 | 70,02 |
| 62 | Visser | 7 | diet | N | 34 | 26,5 | 80,7 | 218,00 | 37,00 | 181 | 122,0 | 70,03 | 117,00 | 69,03 |
| 63 | Gomi | 7 | diet | H | 12 | 51,8 | 59,3 | 94,40 | 24,60 | 69,8 | 145,7 | 93,30 | 144,60 | 93,60 |
| 64 | Facchini | 5 | ss | N | 19 | 43 | 79,4 | 177,60 | 9,60 | 168,7 | 109,2 | 68,10 | 108,90 | 67,90 |
| 65 | Pech.-Bertch | 7 | diet | N | 35 | 28,9 | 60,0 | 224,50 | 46,70 | 177,8 | 109,2 | 75,20 | 106,80 | 74,50 |
| 66 | Pech.-Bertch | 7 | diet | N | 27 | 26 | 59,7 | 217,90 | 25,10 | 192,8 | 108,0 | 71,00 | 107,00 | 73,00 |
| 67 | Ho | 14 | ss | N | 25 | 48,8 | 85,7 | 268,00 | 61,10 | 206,9 | 127,0 | 77,00 | 122,00 | 75,20 |
| 68 | Gijsbers | 28 | ss | H | 36 | 65,8 | 82,5 | 202,90 | 105,10 | 97,6 | 145,3 | 80,60 | 137,80 | 77,90 |
| 69 | McManus | 5 | ss | N | 60 | 50,1 | NA | 199,80 | 97,10 | 102,7 | 126,7 | 76,00 |  |  |
| 70 | Gu | 6 | diet | N | 16 | 28,8 | 69,3 | 283,20 | 59,90 | 223,3 | 117,7 | 70,80 | 118,10 | 73,90 |
| 71 | Selvarajah | 7 | ss | N | 48 | 30 | 68,9 | 224,50 | 73,20 | 151,3 | 120,0 | 73,00 | 116,86 | 71,40 |
| 72 | Matthews | 7 | diet | N | 20 | 40,5 | 79,5 | 229,00 | 24,70 | 204,3 | 120,0 | 74,5 | 115,00 | 71,60 |
| 73 | Jackson | 5 | ss | H | 12 | 52 | NA | 100,00 | 37,00 | 63 | 168,0 | 103 | 156,00 | 95,30 |
| 74 | Sagnella | 5 | ss | N | 11 | 22 | 69 | 296,80 | 22,90 | 273,9 | 118,4 | 73 | 117,80 | 74,20 |
| 75 | Gutkowska | 4 | diet | N | 6 | 29,3 | 68,2 | 254,20 | 46,10 | 213,1 |  |  |  |  |
| 76 | Mallamci | 7 | diet | N | 7 | 34 | 81,3 | 214,00 | 48,00 | 176 | 121,0 |  | 118,00 |  |
| 76 | Mallamaci H | 7 | diet | H | 14 | 47 | 76,4 | 146,00 | 43,00 | 103 | 138,0 |  | 127,00 |  |
| 77 | Naomi | 7 | ss | H | 9 | 46,9 | 60,2 | 255,60 | 28,20 | 227,4 | 145,1 | 86,9 | 130,30 | 80,20 |
| 78 | Singer | 5 | ss | H | 8 | 52,4 | NA | 311,80 | 18,90 | 292,9 | 164,0 | 106 | 163,00 | 106,00 |
| 79 | Zoccali | 7 | diet | H | 14 | 47 | NA | 184,00 | 39,00 | 145 | 156,0 | 101 | 141,00 | 94,00 |
| 80 | Sharma | 5 | ss | N | 24 | 25,5 | 77,3 | 225,60 | 22,00 | 203,6 | 117,8 | 65,5 | 111,50 | 64,10 |
| 81 | Toering | 7 | diet | N | 36 | 33,5 | NA | 211,00 | 40,00 | 171 | 119,5 | 72 | 116,00 | 69,50 |
| 82 | McCubbin | 3 | ss | N | 15 | 40 | 77,1 | 153,00 | 51,00 | 102 |  |  |  |  |
| 83 | Braconnier | 5 | ss | N | 38 | 33,5 | 66,2 | 227,00 | 51,7 | 175,3 | 113,6 | 69,6 | 112,90 | 69,40 |

Dur: Study duration; SS: Slow sodium tablet; BP: Blood pressure; H: Hypertension;

N: Normotension; No: Number; S: Sodium; Hi: High; L: Low; SR: Sodium reduction

**Appendix Figure 1**: Forest plot of conventional meta-analysis of the effect of sodium reduction on aldosterone in normotensive and hypertensive individuals. (Review Manager, Version 5.4, The Cochrane Collaboration, 2020)


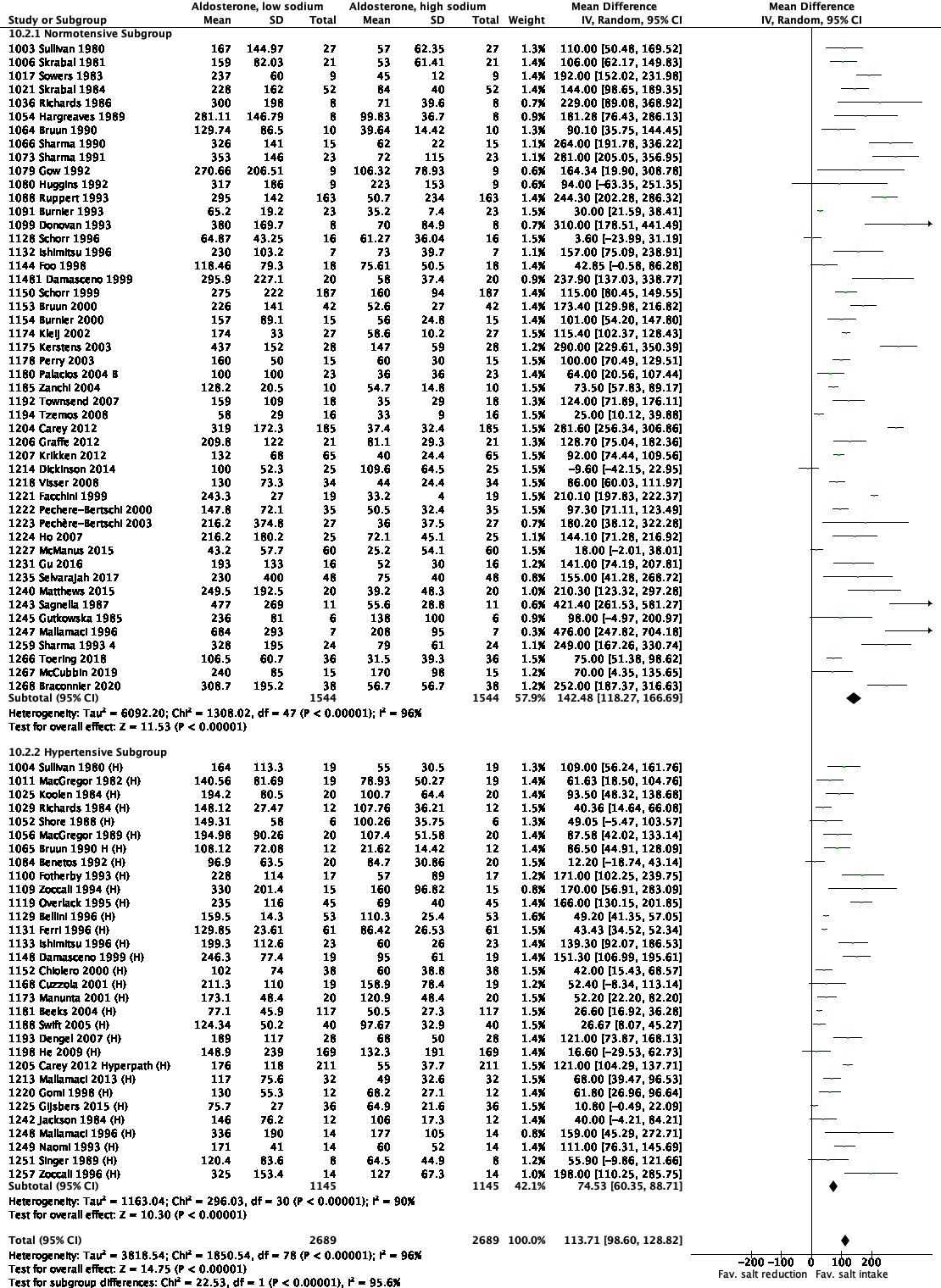


**Appendix Table 2:** Bias Assessment (L: Low risk of bias; H: High risk of bias, U: Unclear risk of bias) (Cochrane risk of bias tool)

| Ref no | 1. Author | Selection | Performance | Attrition | Over-all Bias |
| --- | --- | --- | --- | --- | --- |
| 11 | Sullivan | H | H | L | H |
| 11 | Sullivan H | H | H | L | H |
| 12 | Skrabal | U | H | L | U |
| 13 | Mcgregor | U | L | L | L |
| 14 | Sowers | U | H | L | U |
| 15 | Skrabal | U | H | L | U |
| 16 | Koolen | U | H | L | U |
| 17 | Richards | U | H | H | H |
| 18 | Richards | U | H | L | U |
| 19 | Shore | U | H | L | U |
| 20 | Hargreaves | U | L | L | L |
| 21 | McGregor | U | L | L | L |
| 22 | Brun | U | H | L | U |
| 22 | Brun H | U | H | L | U |
| 23 | Sharma | U | H | U | U |
| 24 | Sharma | U | H | U | U |
| 25 | Gow | U | H | L | U |
| 26 | Huggins | U | L | L | L |
| 27 | Benetos | U | L | L | L |
| 28 | Ruppert | U | H | L | U |
| 29 | Burnier | U | H | L | U |
| 30 | Donovan | U | H | L | U |
| 31 | Fotherby | U | L | L | L |
| 32 | Zoccali | U | H | L | U |
| 33 | Overlack | U | L | L | L |
| 34 | Schorr | U | L | H | U |
| 35 | Bellini | U | L | H | U |
| 36 | Ferri | U | L | L | L |
| 37 | Ishimitsu | U | H | L | U |
| 37 | Ishimitsu H | U | H | L | U |
| 38 | Foo | U | L | L | L |
| 39 | Damasceno | U | L | L | L |
| 39 | Damasceno H | U | L | L | L |
| 40 | Schorr | U | H | L | U |
| 41 | Chiolero | U | H | L | U |
| 42 | Bruun | U | H | L | U |
| 43 | Burnier | U | H | L | U |
| 44 | Cuzzola | U | L | H | U |
| 45 | Manunta | U | U | L | U |
| 46 | Kleij | U | H | L | U |
| 47 | Kerstens | U | H | L | U |
| 48 | Perry | U | H | L | U |
| 49 | Palacios | U | H | H | H |
| 50 | Beeks | U | H | L | U |
| 51 | Zanchi | U | L | L | L |
| 52 | Swift | U | L | U | U |
| 53 | Townsend | U | H | L | U |
| 54 | Dengel | U | L | L | L |
| 55 | Tzemos | U | L | L | L |
| 56 | He W | L | L | L | L |
| 57 | Carey | U | H | U | U |
| 57 | Hyperpath | U | H | U | U |
| 58 | Graffe | L | L | L | L |
| 59 | Krikken | U | H | U | U |
| 60 | Mallamaci | U | H | L | U |
| 61 | Dickinson | U | U | L | U |
| 62 | Visser | U | H | U | U |
| 63 | Gomi | U | H | L | U |
| 64 | Facchini | U | H | L | U |
| 65 | Pechere-Bertchi | U | H | L | U |
| 66 | Pechere-Bertchi | U | H | L | U |
| 67 | Ho | U | H | L | U |
| 68 | Gijsbers | L | L | L | L |
| 69 | McManus | U | H | L | U |
| 70 | Gu | U | H | H | H |
| 71 | Selvarajah | U | L | U | U |
| 72 | Matthews | U | H | U | U |
| 73 | Jackson | L | L | U | L |
| 74 | Sagnella | U | H | U | U |
| 75 | Gutkowska | U | H | U | U |
| 76 | Mallamci | L | H | U | U |
| 76 | Mallamaci H | L | H | U | U |
| 77 | Naomi | U | H | U | U |
| 78 | Singer | U | H | U | U |
| 79 | Zoccali | U | H | U | U |
| 80 | Sharma | U | H | U | U |
| 81 | Toering | U | H | U | U |
| 82 | McCubbin | U | L | U | U |
| 83 | Braconnier | U | H | U | U |

Over-all bias was defined by two or three identical assessments of L (low risk of bias), H (high risk of bias) or U (unclear risk of bias). Three different assessments (1L,1H, 1U) were assessed as U. As renin was measured blindly, detection bias was assumed to be low in all studies

**Appendix Table 3:** Multivariate weighted meta-regression analysis of plasma-aldosterone versus 24-h urinary sodium-excretion, age and DBP (“R” version 3.6.3).

| A: Low salt intake, Normotension, Multiple regression, Aldosterone (weighted) | | | | | |  |
| --- | --- | --- | --- | --- | --- | --- |
|  | Estimate | SE | t | p | 2.5% | 97.5% |
| 24-h sodium low | -1,8284 | 0,5877 | -3,1110 | 0,0035 | -3,0172 | -0,6396 |
| Age | 0,4078 | 1,4991 | 0,2720 | 0,7870 | -2,6244 | 3,4400 |
| DBP (low salt) | -0,7104 | 2,6280 | -0,2703 | 0,7883 | -6,0260 | 4,6051 |
| B: Low salt intake, Hypertension, Multiple regression, Aldosterone (weighted) | | | | | |  |
|  | Estimate | SE | t | p | 2.5% | 97.5% |
| 24-h sodium low | -0,4149 | 0,3275 | -1,2671 | 0,2164 | -1,0880 | 0,2582 |
| Age | -0,3139 | 1,2567 | -0,2498 | 0,8047 | -2,8970 | 2,2693 |
| DBP (low salt) | -0,1903 | 1,3251 | -0,1436 | 0,8869 | -2,9142 | 2,5335 |
| C: Usual/high salt intake, Normotension, Multiple regression, Aldosterone (weighted) | | | | | | |
|  | Estimate | SE | t | p | 2.5% | 97.5% |
| 24-h sodium high | -0,0808 | 0,1121 | -0,7214 | 0,4749 | -0,3073 | 0,1456 |
| Age | -0,3222 | 0,5695 | -0,5658 | 0,5747 | -1,4732 | 0,8287 |
| DBP (high salt) | -0,6255 | 1,0805 | -0,5789 | 0,5659 | -2,8094 | 1,5583 |
| D: Usual/high salt intake, Hypertension, Multiple regression, Aldosterone (weighted) | | | | | | |
|  | Estimate | SE | t | p | 2.5% | 97.5% |
| 24-h sodium high | -0,1453 | 0,0849 | -1,7116 | 0,0989 | -0,3199 | 0,0292 |
| Age | -0,8232 | 0,6328 | -1,3008 | 0,2047 | -2,1239 | 0,4776 |
| DBP (high salt) | 1,6165 | 0,6728 | 2,4025 | 0,0237 | 0,2335 | 2,9996 |
| E: Diff salt intake, Normotension, Multiple regression, Aldosterone difference (weighted) | | | | | | |
|  | Estimate | SE | t | p | 2.5% | 97.5% |
| Sodium reduction | 0,7378 | 0,2102 | 3,5104 | 0,0011 | 0,3130 | 1,1626 |
| Age | 0,0140 | 1,3262 | 0,0106 | 0,9916 | -2,6664 | 2,6944 |
| DBP (high salt) | -2,8871 | 2,4408 | -1,1828 | 0,2439 | -7,8202 | 2,0461 |
| F: Diff salt intake, Hypertension, Multiple regression, Aldosterone difference (weighted) | | | | | | |
|  | Estimate | SE | t | p | 2.5% | 97.5% |
| Sodium reduction | 0,3315 | 0,1188 | 2,7903 | 0,0097 | 0,0873 | 0,5757 |
| Age | 0,4719 | 1,0704 | 0,4408 | 0,6630 | -1,7284 | 2,6721 |
| DBP (high salt) | -1,1696 | 1,1292 | -1,0357 | 0,3099 | -3,4907 | 1,1516 |

**Statistical code**

”R” version 3.6.3: https://www.rdocumentation.org/packages/stats/versions/3.6.2/topics/lm

**References**

1. Graudal N, Hubeck-Graudal T, Jurgens G. Influence of sodium intake and change in sodium intake on plasma-renin in man. EClinicalMedicine. 2021; 33:100750.
2. Singer DR, Markandu ND, Sugden AL, Miller MA, MacGregor GA. Sodium restriction in hypertensive patients treated with a converting enzyme inhibitor and a thiazide. Hypertension 1991; 17: 798-803.
3. Wing LMH, Arnolda LF, Harvey PJ, Upton J, Molloy D, Gabb GM, et al. Low-dose diuretic and/or dieatary sodium restriction when blood pressure is resistant to ACE inhibitor. Blood Pressure 1998; 7: 299-307.
4. Pimenta E, Gaddam KK, Oparil S, Aban I, Husain S, Dell'Italia LJ, et al. Effects of dietary sodium reduction on blood pressure in subjects with resistant hypertension: results from a randomized trial. Hypertension 2009; 54: 475-81.
5. Nowack R, Fliser D, Richter J, Horne C, Mutschler E, Ritz E. Effects of angiotensin-converting enzyme inhibition on renal sodium handling after furosemide injection. Clin Investig 1993;71:622-7.
6. Jula AM, Karanko HM. Effects on left ventricular hypertrophy of long-term nonpharmacological treatment with sodium restriction in mild to moderate essential hypertension. Circulation 1994; 89: 1023-31.
7. Heer M, Baisch F, Kropp J, Gerzer R, Drummer C. High dietary sodium chloride consumption may not induce body fluid retention in humans. Am J Physiol 2000; 278: 585-95.
8. Cavka A, Cosic A, Jukic I, Jelakovic B, Lombard JH, Phillips SA. The role of cyclo-oxygenase-1 in high-salt diet-induced microvascular dysfunction in humans. J Physiol 2015; 593: 5313–24.
9. Jablonski KL, Racine ML, Geolfos CJ, Gates PE, Chonchol M, McQueen MB, et al. Dietary sodium restriction reverses vascular endothelial dysfunction in middle-aged/older adults with moderately elevated systolic blood pressure. J Am Coll Cardiol 2013; 61: 335-43.
10. Paulsen L, Holst LM, Bech JN, Starklint J, Pedersen EB. Glomerular filtration rate and blood pressure are unchanged by increased sodium intake in atorvastatin-treated healthy men. Scand J Clin Lab Invest 2009; 69: 323-9.
11. Sullivan JM, Ratts TE, Taylor JC, Kraus DH, Barton BR, Patrick DR, et al. Hemodynamic effects of dietary sodium in man. Hypertension 1980; 2: 506-14.
12. Skrabal F, Auböck J, Hörtnagel H. Low sodium/high potassium diet for prevention of hypertension: probable mechanisms of action. Lancet 1981; 2(8252): 895-900.
13. MacGregor GA, Markandu ND, Best FE, Elder DM, Cam JM, Sagnella GA, et al. Double-blind randomised crossover trial of moderate sodium restriction in essential hypertension. Lancet 1982; 1(8268): 351-5
14. Sowers JR, Martin VI, Beck FW. Effects of dietary sodium on circadian rhythm and physiological responses of 18-hydroxycorticosterone. Clin Sci 1983; 64: 295-301.
15. Skrabal F, Herholz H, Neumayr M, Hamberger L, Ledochowski M, Sporer H, et al. Salt sensitivity in humans is linked to enhanced sympathetic responsiveness and to enhanced proximal tubular reabsorption. Hypertension 1984; 6: 152-8.
16. Koolen MI, Brummelen P. Sodium sensitivity in essential hypertension: Role of the renin-angiotensin-aldosterone system and the predictive value of an intravenous frusemide test. J Hypertens 1984; 2: 55-9.
17. Richards AM, Nicholls MG, Espiner EA, Ikram H, Maslowski AH, Hamilton EJ, Wells JE. Blood pressure response to moderate sodium restriction and to potassium supplementation in mild essential hypertension. Lancet 1984; 1(8380): 757-61. .
18. Richards AM, Tonolo G, Cleland JG, Leckie BJ, McIntyre GD, Ingram M, et al. Plasma atrial natriuretic peptide: responses to modest and severe sodium restriction. J Hypertens Suppl 1986; 4: S559-63.
19. Shore AC, Markandu ND, McGregor GA. A randomised cross-over study to compare the blood pressure response to sodium loading with and without chloride in patients with essential hypertension. J Hypertens 1988; 6: 613-7.
20. Hargreaves M, Morgan TO, Snow R, Guerin M. Exercise tolerance in the heat on low and normal salt intake. Clin Sci 1989; 76: 553-7.
21. McGregor GA, Markandu ND, Sagnella GA, Singer DRJ, Cappucio FP. Double-blind study of three sodium intakes and long-term effects of sodium restriction in essential hypertension. Lancet 1989; 2(8674): 1244-7.
22. Bruun NE, Skøtt P, Nielsen MD, Rasmussen S, Schütten HJ, Leth A, et al. Normal renal tubular response to changes of sodium intake in hypertensive man. J Hypertens1990; 8: 219-27.
23. Sharma AM, Kribben A, Schattenfroh S, Cetto C, Distler A. Salt sensitivity in humans is associated with abnormal acid-base regulation. Hypertension 1990; 16: 407-13.
24. Sharma AM, Ruland K, Spies KP, Distler A. Salt sensitivity in young normotensive subjects is associated with a hyperinsulinemic response to oral glucose. Journal of Hypertension 1991;9:329-35.
25. Gow IF, Dockrell M, Edwards CRW, Elder A, Grieve J, Kane G, et al. The sensitivity of human blood platelets to the aggregation agent ADP during different dietary sodium intakes in healthy men. Eur J Clin Pharm 1992; 43: 635-8.
26. Huggins RL, Di Nicolantonio R, Morgan, TO. Preferred salt levels and salt taste acuity in human subjects after ingestion of untasted salt. Appetite 1992; 18: 111-9.
27. Benetos A, Xiao YY, Cuche JL, Hannaert P, Safar M. Arterial effects of salt restriction in hypertensive patients. A 9-week, randomized, double-blind, crossover study. J Hypertens 1992; 10: 355-60.
28. Ruppert M, Overlack A, Kolloch R, Kraft K, Göbel B, Stumpe KO. Neurohormonal and metabolic effects of severe and moderate salt restriction in non-obese normotensive adults. Hypertension 1993; 11: 743-9.
29. Burnier M, Rutschmann B, Nussberger J, Versaggi J, Shahinfar S, Waeber B, et al. Salt dependent renal effects of an angiotensin II antagonist in healthy subjects. Hypertension 1993; 22: 339-47.
30. Donovan DS, Solomon CG, Seely EW, Williams GH, Simonson DC. Effect of sodium intake on insulin sensitivity. Am J Physiol 1993; 264: E730-4
31. Fotherby MD, Potter JF. Effects of moderate sodium restriction on clinic and twenty-four-hour ambulatory blood pressure in elderly hypertensive subjects. J Hypertens 1993; 11: 657-63.
32. Zoccali C, Mallamaci F, Parlongo S. The influence of salt intake on plasma calcitonin gene-related peptide in subjects with mild essential hypertension. J Hypertens 1994; 12: 1249-53
33. Overlack A, Ruppert M, Kolloch R, Kraft K, Stumpe KO. Age is a major determinant of the divergent blood pressure responses to varying salt intake in essential hypertension. Am J Hypertens 1995; 8: 829-36.
34. Schorr U, Distler A, Sharma AM. Effect of sodium chloride- and sodium bicarbonate-rich mineral water on blood pressure and metabolic parameters in elderly normotensive individuals: a randomized double-blind crossover trial. J Hypertens 1996; 14: 131-5.
35. Bellini C, Ferri C, Carlomagno A, Necozione S, Lepore A, Desideri G, et al. Impaired inactive to active kallikrein conversion in human salt-sensitive hypertension. JASN 1996; 7: 2565-77
36. Ferri C, Bellini C, Carlomagno A, Desideri G, Santucci A. Active kallikrein response to changes in sodium-chloride intake in essential hypertensive patients. JASN 1996; 7: 443-53.
37. Ishimitsu T, Nishikimi T, Matsuoka H, Kangawa K, Kitamura K, Minami J, et al. Behaviour of adrenomedullin during acute and chronic salt loading in normotensive and hypertensive subjects. Clin Sci 1996; 91: 293-8.
38. Foo M, Denver AE, Coppack SW, Yudkin JS. Effect of salt-loading on blood pressure, insulin sensitivity and limb blood flow in normal subjects. Clin Sci (London) 1998; 95: 157-64
39. Damasceno A, Santos A, Serrao P, Caupers P, Soares-da-Silva P, Polonia J. Deficiency of renal dopaminergic-dependent natriuretic response to acute sodium load in black salt-sensitive subjects in contrast to salt-resistant subjects. J Hypertens 1999; 17: 1995-2001.
40. Schorr U, Blaschke K, Beige J, Distler A, Sharma AM. Angiotensinogen M235T variant and salt sensitivity in young normotensive Caucasians. J Hypertens 1999; 17: 475-9.
41. Chiolero, A, Maillard, M, Nussberger, J, Brunner HR. Proximal sodium reabsorption: An independent determinant of blood pressure response to salt. J Hypertens 2000; 36: 631-7.
42. Bruun NE, Dige-Pedersen H, Skott P. Normal responses of atrial natriuretic factor and renal tubular function to sodium loading in hypertension-prone humans. Blood Pressure 2000; 9: 206-13.
43. Burnier M, Monod M, Chiolero A, Maillard M, Nussberger J, Brunner HR. Renal sodium handling in acute and chronic salt loading/depletion protocols: the confounding influence of acute water loading. J Hypertens 2000; 18: 1657-64.
44. Cuzzola F, Mallamaci F, Tripepi G, Parlongo S, Cutrupi S, Cataliotti A, et al. Urinary adrenomedullin is related to ET-1 and salt intake in patients with mild essential hypertension. Am J Hypertens 2001; 14: 224-30
45. Manunta P, Messaggio E, Ballabeni C, Sciarrone MT, Lanzani C, Ferrandi M, et al. Plasma ouabain-like factor during acute and chronic changes in sodium balance in essential hypertension. Plasma ouabain-like factor during acute and chronic changes in sodium balance in essential hypertension. Hypertension 2001; 38: 198-203
46. van der Kleij FGH, de Jong PE, Henning RH, de Zeeuw D, Navis G. Enhanced responses of blood pressure, renal function, and aldosterone to angiotensin I in the DD genotype are blunted by low sodium intake. JASN 2002; 13: 1025-33.
47. Kerstens MN, van der Kleij FG, Boonstra AH, Sluiter WJ, Koerts J, Navis G, et al. Salt loading affects cortisol metabolism in normotensive subjects: relationships with salt sensitivity. JCEM 2003; 88: 4180-5.
48. Perry CG, Palmer T, Cleland SJ, Morton IJ, Salt IP, Petrie JR, et al. Decreased insulin sensitivity during dietary sodium restriction is not mediated by effects of angiotensin II on insulin action. Clin Sci 2003; 105: 187-94.
49. Palacios C, Wigertz K, Martin BR, Jackman L, Pratt JH, Peacock M, et al. Sodium retention in black and white female adolescents in response to salt intake. JCEM 2004; 89: 1858-63..
50. Beeks E, van der Klauw MM, Kroon AA, Spiering W, Fuss-Lejeune MJMJ, de Leeuw PW. Alpha-adducin Gly460Trp polymorphism and renal hemodynamics in essential hypertension. Hypertension 2004;44:419-23.
51. Zanchi A, Chiolero A, Maillard M, Nussberger J, Brunner HR, Burnier M. Effects of the peroxisomal proliferator-activated receptor-gamma agonist pioglitazone on renal and hormonal responses to salt in healthy men. JCEM 2004; 89: 1140-5.
52. Swift P, Markandu N, Sagnella G, He F, MacGregor GA. Modest salt reduction reduces blood pressure and urine protein excretion in black hypertensives: a randomized control trial. Hypertension 2005; 46: 308-12
53. Townsend RR, Kapoor S, McFadden CB. Salt intake and insulin sensitivity in healthy human volunteers. Clin Sci (London) 2007; 113: 141-8.
54. Dengel DR, Brown MD, Ferrell RE, Reynolds TH, Supiano MA. A preliminary study on T-786C endothelial nitric oxide synthase gene and renal hemodynamic and blood pressure responses to dietary sodium. Physiol Res 2007; 56: 393-401
55. Tzemos N, Lim PO, Wong S, Struthers AD, MacDonald TM. Adverse cardiovascular effects of acute salt loading in young normotensive individuals. Hypertension 2008; 51:1525-35.
56. He FJ, Marciniak M, Visagie E, Markandu ND, Anand V, Dalton RN, et al. Effect of modest salt reduction on blood pressure, urinary albumin, and pulse wave velocity in white, black, and Asian mild hypertensives. Hypertension 2009; 54: 482-8
57. Carey RM, Schoeffel CD, Gildea JJ, Jones JE, McGrath HE, Gordon LN, et al. Salt sensitivity of blood pressure is associated with polymorphisms in the sodium-bicarbonate cotransporter. Hypertension 2012; 60: 1359-66.
58. Graffe CC, Bech JN, Pedersen EB. Effect of high and low sodium intake on urinary aquaporin-2 excretion in healthy humans. Am J Physiol. Ren Physiol. 2012; 15: F264-75.
59. Krikken JA, Dallinga-Thie GM, Navis G, Dullaart RP. Short term dietary sodium restriction decreases HDL cholesterol, apolipoproteinA-I and high molecular weight adiponectin in healthy young men: relationships with renal hemodynamics and RAAS activation. NMCD 2012; 22: 35-41.
60. Mallamaci F, Leonardis D, Pizzini P, Cutrupi S, Tripepi G, Zoccali C. Procalcitonin and the inflammatory response to salt in essential hypertension: a randomized cross-over clinical trial. J Hypertens 2013; 31: 1424-30.
61. Dickinson KM, Clifton PM, Keogh JB. A reduction of 3 g/day from a usual 9g/day salt diet improves endothelial function and decreases endothelin-1 in a randomised cross over study in normotensive overweight and obese subjects. Atherosclerosis 2014; 233: 32-8.
62. Visser FW, Boonstra AH, Titia Lely A, Boomsma F, Navis G. Renal response to angiotensin II is blunted in sodium-sensitive normotensive men. Am J Hypertens 2008; 21: 323-8.
63. Gomi T, Shibuya Y, Sakurai J, Hirawa N, Hasegawa K, Ikeda T. Strict dietary sodium reduction worsens insulin sensitivity by increasing sympathetic nervous activity in patients with primary hypertension. Am J Hypertens 1998; 11: 1048-55.
64. Facchini FS, Do Nascimento C, Reaven GM, Yip JW, Ni XP, Humphreys MH. Blood pressure, sodium intake, insulin resistance, and urinary nitrate excretion. Hypertension 1999; 33: 1008-12
65. Pechère-Bertschi A, Maillard M, Stalder H, Brunner HR, Burnier M. Blood pressure and renal haemodynamic response to salt during the normal menstrual cycle. Clin Sci (London) 2000; 98: 697-702.
66. Pechère-Bertschi A, Maillard M, Stalder H, Bischof P, Fathi M, Brunner HR, et al. Renal hemodynamic and tubular responses to salt in women using oral contraceptives. KI 2003; 64: 1374-80.
67. Ho JT, Keogh JB, Bornstein SR, Ehrhart-Bornstein M, Lewis JG, Clifton PM, et al. Moderate weight loss reduces renin and aldosterone but does not influence basal or stimulated pituitary-adrenal axis function. Hormone and Metabolic Research 2007; 39: 694-9.
68. Gijsbers L, Dower JI, Schalkwijk CG, Kusters YH, Bakker SJ, Hollman PC, et al. Effects of sodium and potassium supplementation on endothelial function: a fully controlled dietary intervention study. BJN 2015; 114: 1419-26
69. McManus F, Fraser R, Davies E, Connell JMC, Freel EM. Plasma steroid profiling and response to trophins to illustrate intra-adrenal dynamics. Clin Endocrinol (Oxford) 2015; 82: 149-57
70. Gu N, Cho JY, Shin KH, Jang IJ, Rhee MY. The influence of dietary sodium content on the pharmacokinetics and pharmacodynamics of fimasartan. Drug Des Devel Ther 2016; 10: 1525-31.
71. Selvarajah V, Mäki-Petäjä KM, Pedro L, Bruggraber SFA, Burling K, Goodhart AK, Brown MJ, McEniery CM, Wilkinson IB. Novel Mechanism for Buffering Dietary Salt in Humans: Effects of Salt Loading on Skin Sodium, Vascular Endothelial Growth Factor C, and Blood Pressure. Hypertension 2017; 70: 930-937.
72. Matthews EL, Brian MS, Ramick MG, Lennon-Edwards S, Edwards DG, Farquhar WB. High dietary sodium reduces brachial artery flow-mediated dilation in humans with salt-sensitive and salt-resistant blood pressure. J Appl Physiol 2015; 118: 1510-5.
73. Jackson B, Cubela R, Johnston CI. Effect of dietary sodium on angiotensin-converting enzyme (ACE) inhibition and the acute hypotensive effect of enalapril. (MK-421) in essential hypertension. J Hypertens 1984;2:371-7.
74. Sagnella GA, Markandu ND, Shore AC, Forsling ML, MacGregor GA. Plasma atrial natriuretic peptide: its relationship to changes in sodium intake, plasma renin activity and aldosterone in man. Clin Sci (London) 1987; 72: 25-30.
75. Gutkowska J, Schiffrin EL, Cantin M, Genest J. Effect of dietary sodium on plasma concentration of immunoreactive atrial natriuretic factor in normal humans. Clin Invest Med. 1986; 9: 222-4.
76. Mallamaci F, Leonardis D, Bellizzi V, Zoccali C. Does high salt intake cause hyperfiltration in patients with essential hypertension? J Hum Hypertens 1996; 10: 157-61.
77. Naomi S, Umeda T, Iwaoka T, Yamauchi J, Ideguchi Y, Fujimoto Y, et al. Endogenous erythropoietin and salt sensitivity of blood pressure in patients with essential hypertension. Am J Hypertens 1993; 6: 15-20.
78. Singer DR, Markandu ND, Buckley MG, Miller MA, Sugden AL, Sagnella GA, et al. Prolonged decrease in blood pressure after atrial natriuretic peptide infusion in essential hypertension: a new anti-pressor mechanism? Clin Sci (Lond). 1989; 77: 253-8.
79. Zoccali C, Mallamaci F, Cuzzola F, Leonardis D. Reproducibility of the response to short-term low salt intake in essential hypertension. J Hypertens 1996; 14: 1455-9.
80. Sharma AM, Cetto C, Schorr U, Spies KP, Distler A. Renal acid-base excretion in normotensive salt-sensitive humans. Hypertension. 1993 ;22:884-90.
81. Toering TJ, Gant CM, Visser FW, van der Graaf AM, Laverman GD, Danser AHJ, et al. Sex differences in renin-angiotensin-aldosterone system affect extracellular volume in healthy subjects. Am J Physiol Renal Physiol 2018; 314: F873-F878.
82. McCubbin AJ, Lopez MB, Cox GR, Caldwell Odgers JN, Costa RJS. Impact of 3-day high and low dietary sodium intake on sodium status in response to exertional-heat stress: a double-blind randomized control trial. Eur J Appl Physiol. 2019;119:2105-2118.
83. Braconnier P, Milani B, Loncle N, Lourenco JM, Brito W, Delacoste J, et al. Short-term changes in dietary sodium intake influence sweat sodium concentration and muscle sodium content in healthy individuals. J Hypertens 2020; 38: 159-166.
84. Bovée DM, Visser WJ, Middel I, De Mik-van Egmond A, Greupink R, Masereeuw R, et al. A Randomized Trial of Distal Diuretics versus Dietary Sodium Restriction for Hypertension in Chronic Kidney Disease. JASN 2020; 31:650-662.
85. Bovée DM, Roksnoer LCW, van Kooten C, Rotmans JI, Vogt L, de Borst MH, et al. Effect of sodium bicarbonate supplementation on the renin- angiotensin system in patients with chronic kidney disease and acidosis: a randomized clinical trial. J Nephrol 2020 Dec 31. doi:10.1007/s40620-020-00944-5. Epub ahead of print.
86. Baqar S, Kong YW, Chen AX, O'Callaghan C, MacIsaac RJ, Bouterakos M, et al. Effect of Salt Supplementation on Sympathetic Activity and Endothelial Function in Salt-Sensitive Type 2 Diabetes. JCEM 2020; 105: dgz219.
87. Toft U, Riis NL, Lassen AD, Trolle E, Andreasen AH, Frederiksen AKS, et al. The Effects of Two Intervention Strategies to Reduce the Intake of Salt and the Sodium-To-Potassium Ratio on Cardiovascular Risk Factors. A 4-Month Randomised Controlled Study among Healthy Families. Nutrients 2020; 12: 1467.
88. Pechère-Bertschi A, Olivier V, Burnier M, Udwan K, de Seigneux S, Ponte B, Maillard M, Martin PY, Feraille E. Dietary sodium intake does not alter renal potassium handling and blood pressure in healthy young males. Nephrol Dial Transplant 2022; **37:** 548-57.
89. Graudal NA, Hubeck-Graudal T, Jurgens G. Effects of low sodium diet versus high sodium diet on blood pressure, renin, aldosterone, catecholamines, cholesterol, and triglyceride. Cochrane Database Syst Rev. 2020;12 (12):CD004022.
